# Supplementary material for: Interaction of Pelargonium sidoides Compounds with Lactoferrin and SARS-CoV-2: Insights from Molecular Simulations
Source: Int J Environ Res Public Health. 2022 Apr 26;19(9):5254. doi: 10.3390/ijerph19095254 (PMC9101775; doi:10.3390/ijerph19095254)
Supplement: Supplementary file 1 [file ijerph-19-05254-s001.zip › ijerph-1644021-supplementary.pdf]

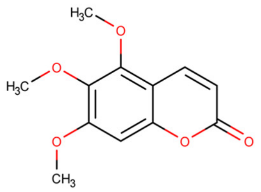

5,6,7-trimethoxycoumarin

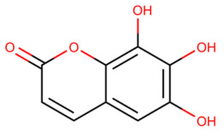

6,7,8-trihydroxycoumarin

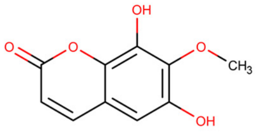

6,8-dihydroxy-7 methoxycoumarin

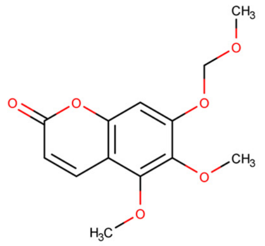

7-acetoxy-5,6-dimethoxycoumarin

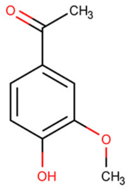

Apocynin

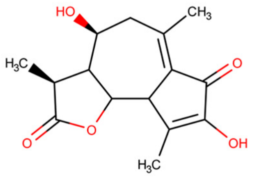

Artelin

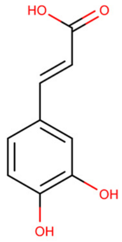

Caffeic acid

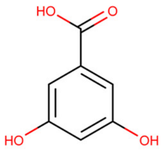

Dihydroxybenzoic acid

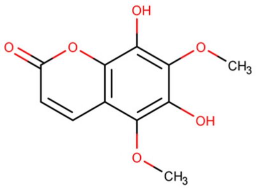

Dimethoxycoumarin

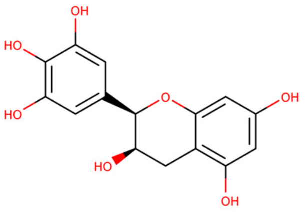

Epigallocatechin-3-gallate

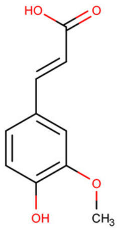

Ferulic acid

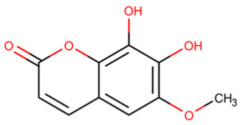

Fraxetin

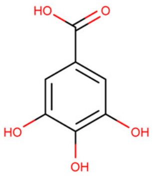

Gallic acid

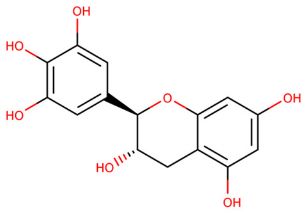

Gallocatechin

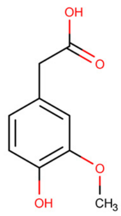

Homovanillic acid

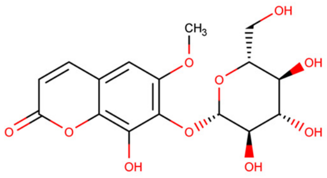

Isofraxoside

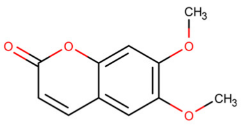

Isoorientin

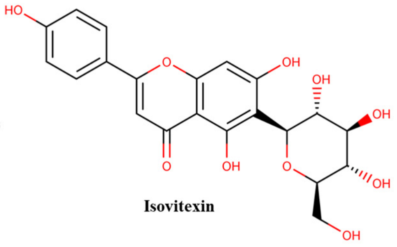

Isovitexin

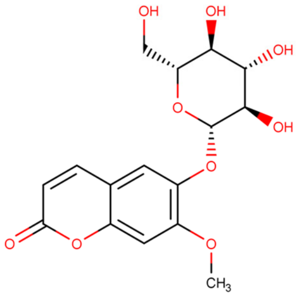

Magniolioside

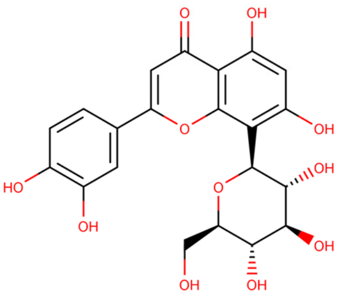

Orientin

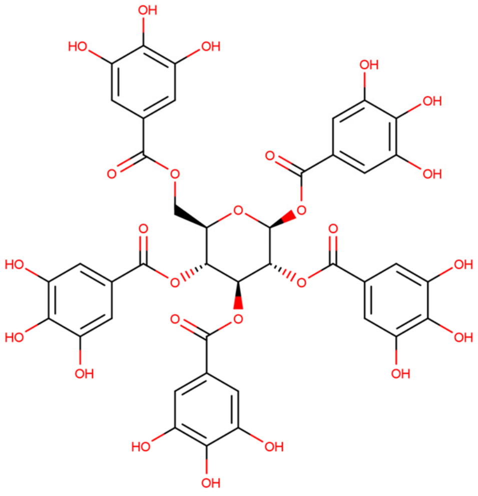

Pentagalloylglucose

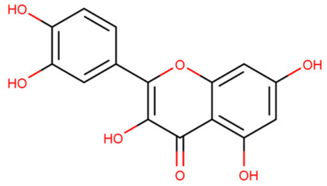

Quercetin

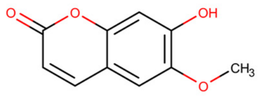

Scopoletin

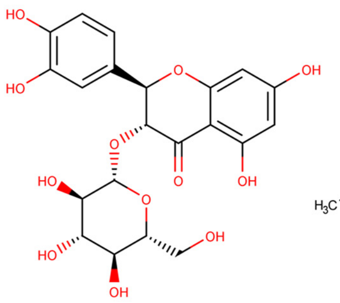

Taxifolin-3-glucoside

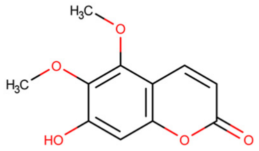

Umckalin

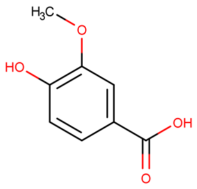

Vanillic acid

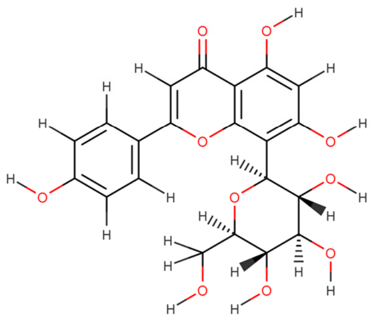

Vitexin

Figure S1. Schematic representation of chemical structure of the PEL compounds used for molecular docking and molecular dynamics simulations.

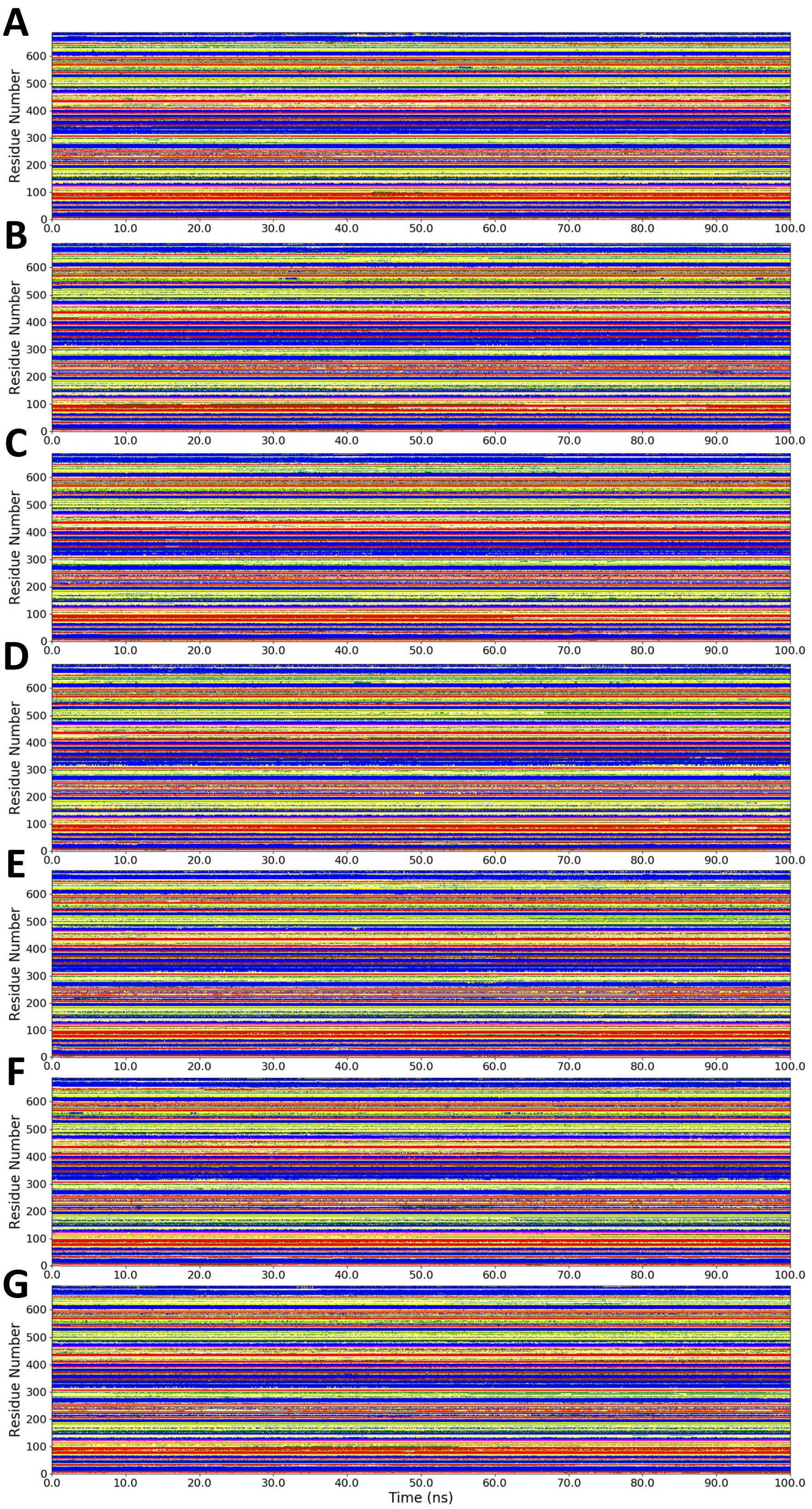

Figure S2. Secondary structure time evolution calculated for the bovine lactoferrin in complex with epigallocatechin **(A)**, Taxifolin-3-glucoside **(B)**, Gallocatechin **(C)**, Artelin **(D)**, Pentagalloyl glucose **(E)**, Vitexin **(F)** and Isoorientin **(G).** Color legend identifying the secondary structure elements is shown below.

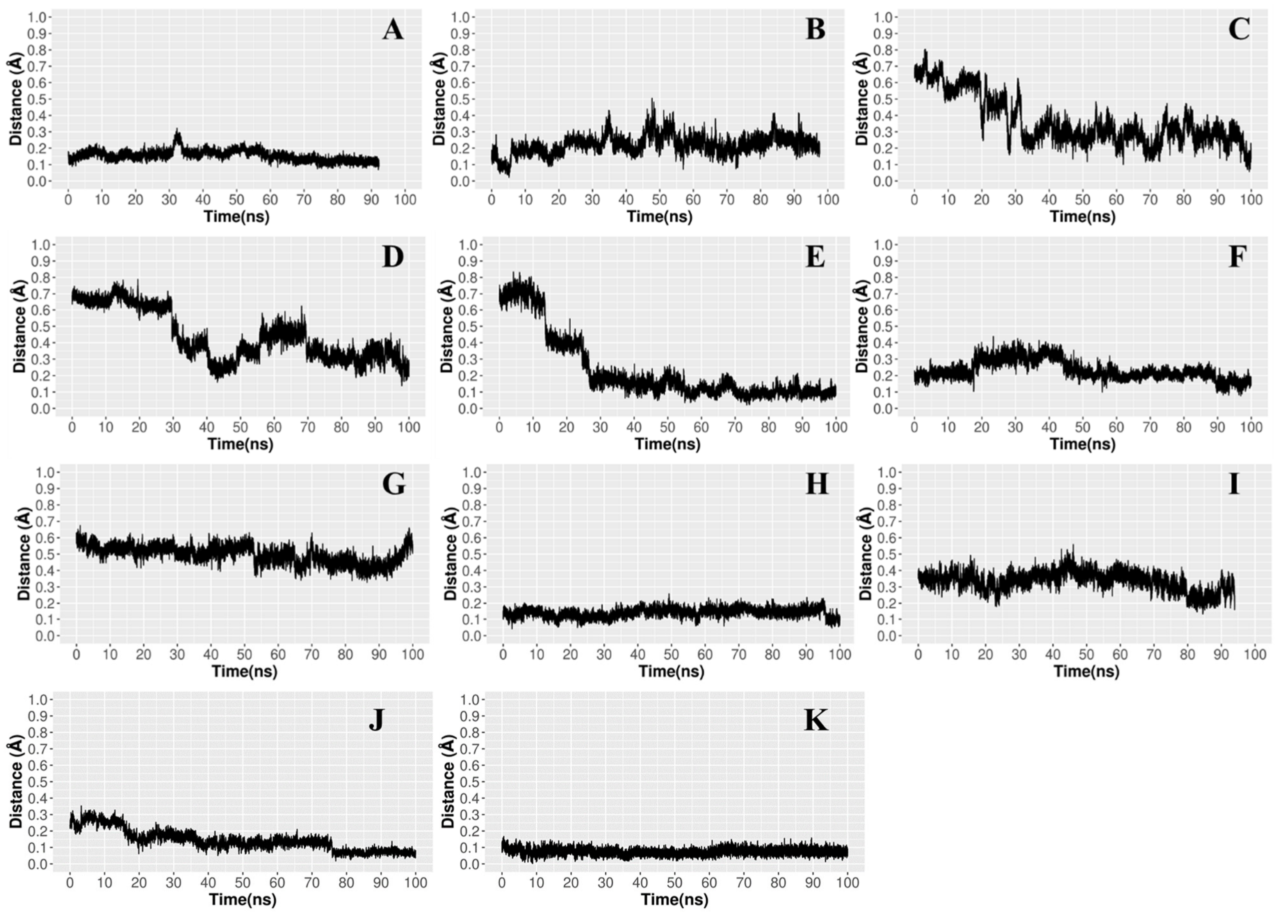

Figure S3. Distance as a function of simulation time calculated between the HR1 site of the spike glycoprotein and the compounds identified through docking. (**A**) Complex between spike glycoprotein and Pentagalloyl glucose. (**B**) Complex between spike glycoprotein and Vitexin. (**C)** Complex between spike glycoprotein and 6-8-dihydroxy-7-methoxycoumarin. (**D**) Complex between spike glycoprotein and Quercetin. (**E**) Complex between spike glycoprotein and Taxifolin-3-glucoside. (**F**) Complex between spike glycoprotein and Isofraxoside. (**G**) Complex between spike glycoprotein and Isovitexin. (**H**) Complex between spike glycoprotein and Gallocatechin. (**I**) Complex between spike glycoprotein and Isoorientina. (**J**) Complex between spike glycoprotein and Epigallocatechin. (**K**) Complex between the spike glycoprotein and the Magnolioside.

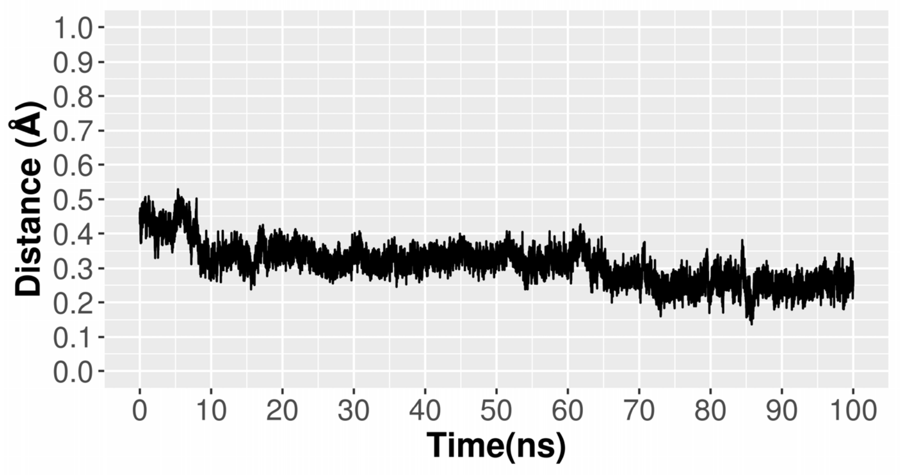

Figure S4. Distance as a function of simulation time calculated between the docking-identified site on the RdRp polymerase protein and the pentagalloyl glucose.

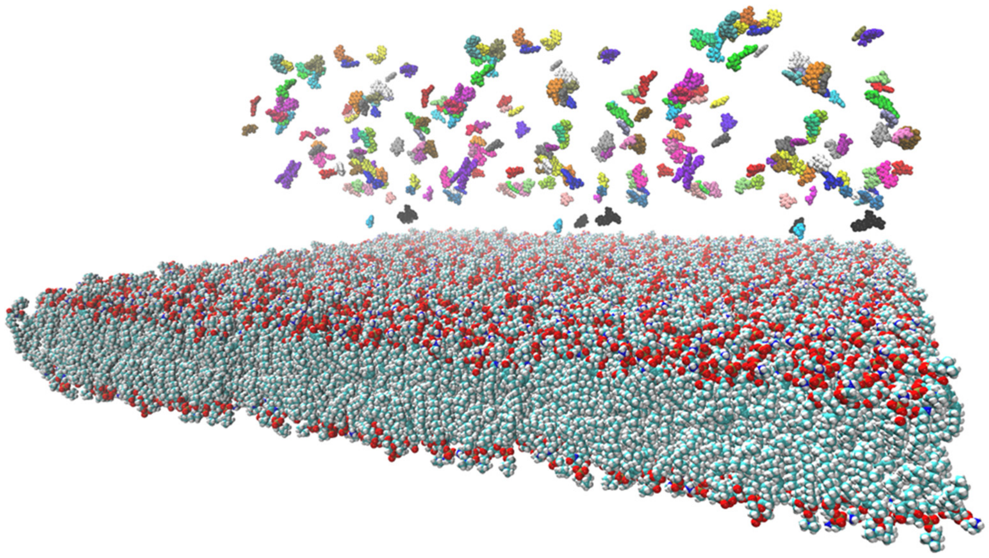

Figure S5. Molecular space-fill representation of the membrane-PEL compounds system. The different colors of the spheres highlight the 27 different compounds.

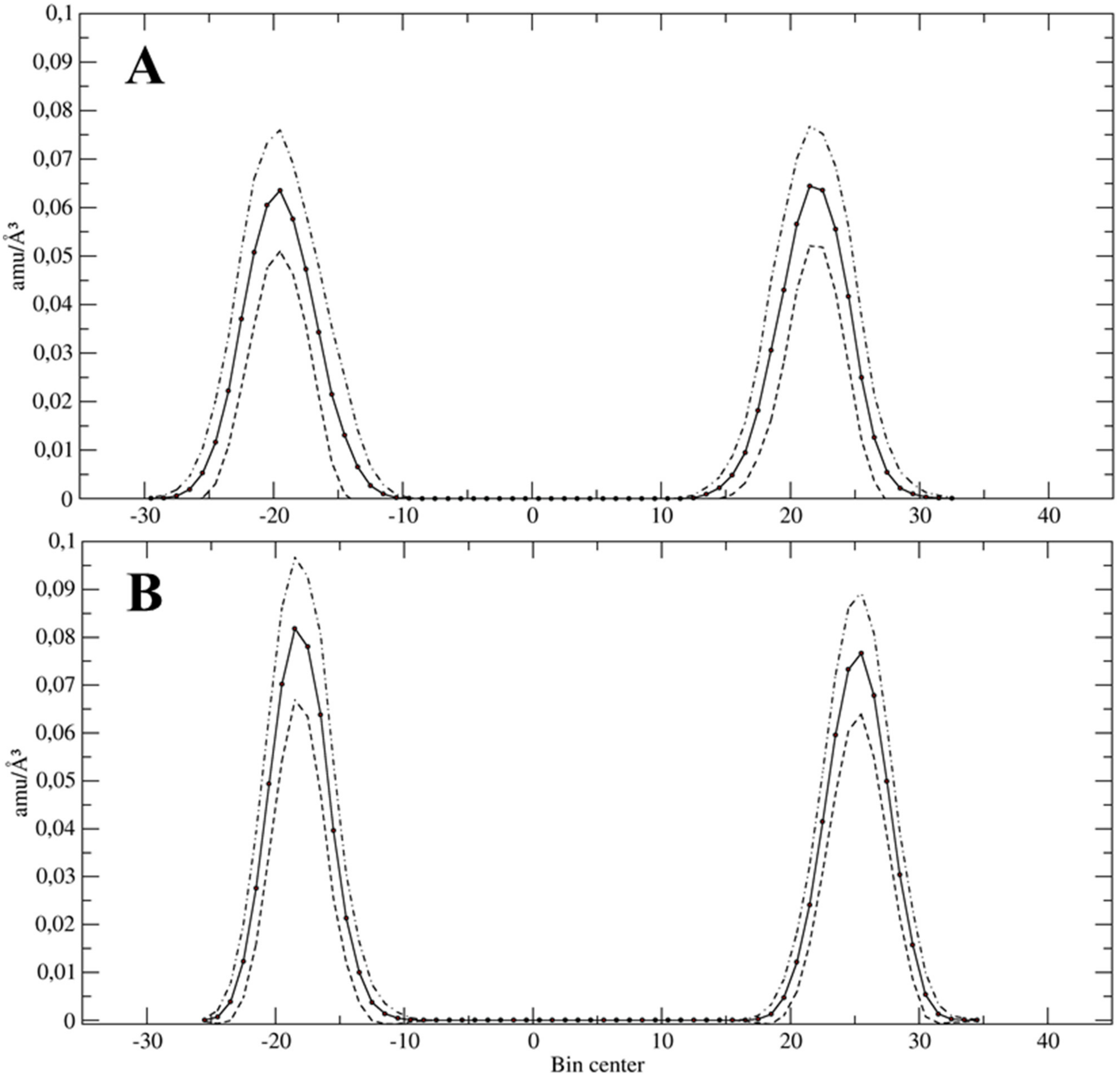

Figure S6. Density profiles of the two membrane layers as a function of the z axis for the system simulated in the absence (**A**) and presence (**B**) of the PEL compounds.

Table S1. Prediction of oral acute toxicity, class, and toxicity model of PEL compounds. Class I: death after swallowing (LD50 ≤ 5); Class II: death after swallowing (5< LD50 ≤50); Class III: toxic after swallowing (50 < LD50 ≤ 300); Class IV: harmful after swallowing (300<LD50≤2000); Class V: may be harmful after swallowing (2000 <LD50 ≤5000) and Class VI: non-toxic (LD50 > 5000).

Compound	ProTox-II Toxicity Class	ProTox-II LD50 (mg/kg)	ProTox-II Toxicity Model Report
6,7,8-trihydroxycoumarin	IV	2991	Putative immunotoxicity
5,6,7-trimethoxycoumarin	V	3800	Immunotoxicity (IL1RL1 inhibitor)
6,8-dihydroxy-7 methoxycoumarin	V	3800	Putative immunotoxicity
Apocynin	VI	9000	-
7-acetoxy-5,6-dimethoxycoumarin	V	3800	Putative immunotoxicity
Artelin	V	3800	-
Dihydroxybenzoic acid	IV	1800	-
Caffeic acid	V	2980	Putative carcinogenicity
Ferulic acid	IV	1772	Putative immunotoxicity
Epigallocatechin	VI	10000	-
Dimethoxycoumarin	V	3800	-
Gallic acid	IV	2000	-
Fraxetin	V	3800	Putative immunotoxicity
Isofraxoside	V	5000	Putative immunotoxicity
Homovanillic acid	IV	1123	-
Gallocatechin	VI	10000	-
Magnolioside	V	4000	-
Isovitexin	III	159	-
Isoorientin	III	159	-
Pentagalloyl-glucose	V	2260	AhR activation
Taxifolin-3-glucoside	V	2300	-
Scopoletin	V	3800	-
Quercetin	III	159	Estrogen receptor α modulation
Vanillic acid	IV	2000	-
Umckalin	V	3800	-
Vitexin	IV	832	-
